# Nucleotide sequence characterization, amino acid variations and 3D structural analysis of HN protein of the NDV VIId genotype

**DOI:** 10.1002/vms3.1491

**Published:** 2024-06-21

**Authors:** Amin Tavassoli, Safoura Soleymani, Mohammad Reza Housaindokht

**Affiliations:** ^1^ Research and Technology Center of Biomolecules Faculty of Science, Ferdowsi University of Mashhad Mashhad Iran; ^2^ Department of Chemistry Faculty of Sciences, Ferdowsi University of Mashhad Mashhad Iran

**Keywords:** antigenic regions, bioinformatics, haemagglutinin–neuraminidase protein, Newcastle disease virus, substitutions, VIId subgenotype

## Abstract

**Background:**

Haemagglutinin–neuraminidase (HN) is one of the membrane proteins of Newcastle disease virus (NDV) that plays a significant role during host viral infection. Therefore, antibodies against HN are vital for the host's ability to protect itself against NDV infection due to their critical functions in viral infection. As a result, HN has been a candidate protein in vaccine development against the Newcastle disease virus.

**Methods:**

This report used the full‐length sequence of the HN protein of NDV isolated in Iran (VIId subgenotype). We characterize and identify amino acid substitutions in comparison to other more prevalent NDV genotypes, VII subgenotypes and vaccine strains. Furthermore, bioinformatics tools were applied to determine the three‐dimensional structure, molecular dynamics simulation and prediction of B‐cell antigenic epitopes.

**Results:**

The results showed that the antigenic regions of our isolate are quite comparable to the other VII subgenotypes of NDV isolated from different geographical places. Moreover, by employing the final 3D structure of our HN protein, the amino acid residues are proposed as a B‐cell epitope by epitope prediction servers, which leads to the introduction of linear and conformational antigenic sites.

**Conclusions:**

Immunoinformatic vaccine design principles currently exhibit tremendous potential for developing a new generation of candidate vaccines quickly and economically to eradicate infectious viruses, including the NDV. In order to accomplish this, focus is directed on residues that might be considered antigenic.

## Graphical abstract

Graphical Abstract:

• Nucleotide and amino acid sequence characterization of NDV HN protein.

• Three‐dimensional and molecular dynamics simulation analysis.

• Applying immunoinformatic analysis for finding linear and conformational antigenic epitopes.

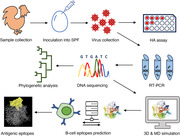

## 1 INTRODUCTION

Newcastle disease (ND) is a highly contagious and economically significant disease for many avian species worldwide. The causative agent of ND is virulent strains of Newcastle disease virus (NDV), which cause numerous economic losses in the poultry industry, including a high percentage of morbidity and mortality along with decreased egg production (Berhanu et al., 2010; Sultan et al., 2020; Yan et al., 2020). Based on their pathogenicity, the NDV strains are categorized into three main pathotypes: velogenic (highly pathogenic), mesogenic (moderately pathogenic) and lentogenic (non‐ or mildly pathogenic) (Jin et al., 2017; Samal et al., 2013; Sultan et al., 2020). NDV is an enveloped virus with a non‐segmented, single‐stranded RNA genome and belongs to the genus Avulavirus within the family Paramyxoviridae (Berhanu et al., 2010; Bose et al., 2014; Cho et al., 2007). The viral RNA genome encodes six major structural proteins: nucleocapsid (N), matrix (M), phosphoprotein (P), fusion (F), haemagglutinin–neuraminidase (HN) and large polymerase (L), of which only the F and HN glycoproteins interact with the viral envelope and are significant contributors to pathogenic and antigenic features, as well as key players in determining the NDV strain host range (Cho et al., 2007; Huang et al., 2004; Yan et al., 2020). Moreover, it demonstrated that these two glycoproteins on the external surface of the viral envelope elicit neutralizing antibodies to protect hosts against NDV (Collins et al., 1993; Liu et al., 2015; Sabouri et al., 2018).

The F protein facilitates viral penetration into cells by fusing the viral envelope with the host cell membrane (Berhanu et al., 2010, Bose et al., 2014). Host proteases have identified the amino acid sequence at the F protein cleavage site as the major determinants of NDV tissue tropism and virulence (Huang et al., 2004; Jin et al., 2017). Because different amino acid sequences are present at this site in different virus strains, the number of these basic residues determines the type of protease enzymes that affect them. In virulence strains, the cleavage sites with multiple basic amino acids are cleaved in most cell types, leading to the systemic spread of viruses. In contrast, in avirulent strains based on the type of secreted protease, the virus's spread is usually restricted to the mucosal surfaces of the respiratory and enteric tracts (OIE, 2012; Sabouri et al., 2018; Samal et al., 2013). HN is another critical virulence factor of NDV. It acts as a multifunctional surface glycoprotein and mediates various activities, such as interacting with the F protein to promote membrane fusion and virus penetration (Estevez et al., 2011; Huang et al., 2004; Khattar et al., 2009; Liu et al., 2015). In addition, haemagglutination assay (HA) causes erythrocyte aggregation and attachment to sialic acid‐containing receptor(s) on cell surfaces as well as actings on neuraminidase (NA) to release sialic acid from progeny virus particles, preventing viral self‐aggregation. HA and NA activities are specified functions for HN protein (Estevez et al., 2011; Huang et al., 2004; Khattar et al., 2009).

In NDV strains, HN is a type II homotetrameric glycoprotein with multiple functions that has an amino acid length between 570 and 616 (Jakhesara et al., 2016; Khattar et al., 2009; Liu et al., 2019). This protein structurally consists of a cytoplasmic tail domain, a transmembrane domain, a stalk domain and a globular head domain. The globular head contains receptor‐binding, antigenic and neuraminidase‐active sites. In addition, the X‐ray crystal structure of the globular head domain's core structure, known as the propeller, revealed residues that contribute to neuraminidase, receptor‐binding and fusion activities (Khattar et al., 2009; Liu et al., 2019; Yan et al., 2020; Yuan et al., 2011; Zhu et al., 2016).

Although there are not much publications on the molecular characteristics of this gene among Iranian NDVs, it is well known that HN protein plays a key role as a surface glycoprotein during the attachment, assembly, maturation and virulence of NDVs. Despite the continuous implementation of vaccination programs, NDV outbreaks are still a significant problem in the Iranian poultry industry and frequently occur in Iran and other Asian countries (Sabouri et al., 2018; Triosanti et al., 2018; Zhu et al., 2016). Therefore, it is impressive to find this protein's genomic and antigenic characteristics that might contribute to ND control and might be the way forward to developing a new generation of vaccines to eradicate this infectious disease.

One of the most effective approaches to control ND is proper vaccination to prevent virus spread. The most widely used ND vaccines belong to genotypes I and II, which were isolated nearly 70 years ago. Nevertheless, the prevalent NDV strains in poultry belong to genotypes V–VII on different continents, which are genetically distinct from current vaccines. Many studies have consistently verified that traditional ND vaccines cannot reduce virus shedding from vaccinated chickens (Kapczynski & King, 2005; Kim et al., 2017). Due to the low similarity antigens between conventional vaccines and prevalent strains, the investigation and development of safe, genotype‐matched and efficient ND vaccines is an important need (Hu et al., 2022). New vaccine development strategies are focused on recombinant protein vaccines, antigenically matched vaccines and genetically attenuated live vaccines.

In the previous study (Tavassoli et al., 2019), we have shown that three isolates of NDV collected from clinical specimens in northeastern Iran during 2014–2016 were velogenic on the basis of both the ICPI and MDT values and the phylogenetic tree of the nucleotide sequence of the F gene. In this study, due to the similarity of the sequences of isolates, we used one isolate to investigate the full‐length sequence of the HN protein of the NDV isolated. We characterize and identify amino acid substitutions compared with other more prevalent NDV genotypes, VII subgenotypes and vaccine strains at the receptor binding, fusion promotion, sialic acid binding and antigenic sites for comparison with other more prevalent NDV genotypes, VIId subgenotypes and vaccine strains. Moreover, bioinformatics tools are employed to determine the 3D structure, molecular dynamics simulation and prediction of B‐cell antigenic epitopes. It seems that the characterization of this recent isolate may help to gain invaluable information about the pathogenicity and epidemiological relationships of Asian outbreaks, especially in Iran, with the other NDV strains in the world.

## 2 MATERIALS AND METHODS

### 2.1 Sample collection, virus propagation and HA testing

The isolate reported in this study was collected from the trachea and intestines of dead birds in northeastern Iran in 2016. The birds displayed clinical signs, including drooping wings, paralysis of the legs and twisting the head and neck. In addition, post‐mortem lesions revealed petechiae in the proven triculus and haemorrhagic ulcers in the intestinal wall. The tissue materials, such as trachea and intestine showing petechiae, were homogenized in PBS solution, filtered through a 0.2 µm pore size filter and inoculated into the allantoic cavity of 10‐day‐old embryonated SPF eggs and incubated at 37°C. The infected allantoic fluids were harvested 2 days post‐inoculation and analysed for NDV by HA using 1% chicken red blood cells at room temperature and stored at freezer (−80°C) until further processing.

### 2.2 Isolation of viral RNA, RT‐PCR, sequencing and phylogenetic analysis

Viral genomic RNA was extracted from virus‐infected allantoic (Total RNA purification kit, Jena Bioscience) and subjected to cDNA synthesis (AccuPower RT PreMix, Bioneer). Thermo Scientific's Phusion High‐Fidelity PCR Kit was used to amplify the HN's entire coding sequence following the manufacturer's instructions. The PCR primers (Table 1) used in this study were designed by employing the consensus sequences of HN genes from the GenBank database of the NCBI. Then the PCR products encoding the HN gene were sequenced in both directions by Sanger sequencing (Macrogen Co.). Next, the sequences were assembled and analysed by BioEdit software. Finally, the open reading frame of the HN gene was submitted to GenBank for the isolate sequenced in this study with a specified accession number of MN327574.1.

**TABLE 1:** List of primers used in this study.

Position	Product (bp)	Sequence (5′–3′)	Primer name
**5924–5945**	754	ATTCTCAAGTCATCGTGACAGG	NDV‐HN‐F1
**6657–6677**		CACATCTTGACTTGAACTGAG	NDV‐HN‐R1
**6483–6502**	811	GAAGCAAAGAACACATGGCG	NDV‐HN‐F2
**7272–7293**		AGTCCTTCTCATGGTATTGACC	NDV‐HN‐R2
**7107‐7129**	1362	CTGCGCTCCATCAATTTAGATGA	NDV‐HN‐F3
**8446–8468**		GATAGATGTGACTCTGGTAGGAT	NDV‐HN‐R3

The phylogenetic tree and evolutionary distance analysis are employed to determine the genotype and subgenotype of our isolate. For these purposes, we retrieved the nucleic acid sequences for the HN gene of widely distributed NDV viruses worldwide from the nucleotide database. Table 2 contains a list of GenBank accession numbers and the corresponding country of origin of each nucleotide sequence that we used for phylogenetic analysis. All of the open reading frames of the HN genes were subjected to sequence alignment, editing, predicting coding regions, determining the amino acid substitutions and phylogenetic analysis using Clustal Omega, BioEdit and CLC Main Workbench software. In addition, phylogenetic and molecular evolutionary distances among different NDV virus groups were conducted by the neighbor‐joining algorithm (Kimura 2‐parameter, bootstrapped 1000 replicates) of MEGA software (v. X). For these, the nucleotide sequences of the entire ORF of the HN gene from 1 to 1716 nucleotides were compared.

**TABLE 2:** The Newcastle disease virus (NDV) strains used in the analysis of phylogenetic tree.

Genotype/Subgenotype	Country	Gene Bank NO.	No.	Genotype/Subgenotype	Country	Gene Bank NO.	No.
VIIb	China	JQ015295	41	I	USA	AY562991	1
VIIb	China	KC542899	42	I	Australia	AY935494	2
VIIb	China	JX840450	43	I	Japan	M19478	3
VIIb	China	JQ015296	44	I	Hungary	DQ097394	4
VIIi	Iran	KU201418	45	II	China	AY845400	5
VIIi	Iran	KU201419	46	II	China	JF950510	6
VIIi	China	KJ607169	47	II	USA	EU289028	7
VIIe	China	DQ659677	48	II	USA	AF309418	8
VIIe	China	DQ485265	49	II	USA	AF375823	9
VIIe	China	DQ485263	50	II	Germany	Y18898	10
VIIf	China	GQ338310	51	III	China	FJ430160	11
VIIf	China	JF343538	52	III	China	FJ430159	12
VIIg	China	KC542895	53	III	China	EF201805	13
VIIe	China	DQ485265	54	III	China	GU573804	14
VIIg	China	GQ994433	55	IV	China	EU293914	15
VIIh	Indonesia	HQ697261	56	IV	The Netherlands	AY741404	16
VIIh	Indonesia	HQ697256	57	IV	Hungary	X71994	17
VIII	China	FJ751919	58	V	USA	AY562986	18
VIII	China	FJ751918	59	V	USA	AY288993	19
IX	China	FJ436302	60	V	USA	GQ288386	20
IX	China	FJ436305	61	V	USA	GQ288385	21
X	Iran	GQ288392	62	V	USA	GQ288381	22
X	Iran	GQ288390	63	V	Mexico	HM117720	23
XI	France	HQ266603	64	VI	Hungary	AJ880277	24
XI	France	HQ266602	65	VI	USA	AY562988	25
XI	France	HQ266604	66	VI	The Netherlands	GQ429293	26
XII	Peru	JN800306	67	VI	China	FJ766529	27
XII	Peru	KR732614	68	VI	USA	FJ410145	28
XII	China	JN627511	69	VI	China	FJ766528	29
XII	China	JN627513	70	VIa	China	GQ281092	30
XIIIa	India	KJ577585	71	VIa	USA	AY288996	31
XIIIa	India	KF727980	72	VIId	China	AF431744	32
XIIIa	Russia	AY865652	73	VIId	China	FJ872531	33
XIIIb	India	KF740478	74	VIId	China	AF473851	34
XIIIb	India	KP089979	75	VIId	China	GQ245843	35
XIIIb	Pakistan	JN682211	76	VIId	China	GQ245847	36
XIIIb	Pakistan	JN682210	77	VIId	China	GQ245860	37
XVI	Dominican Republic	JX119193	78	VIId	China	GQ245870	38
Class I	USA	EF520714	79	VIId	China	DQ486859	39
Class I	Japan	AB524405	80	VIIb	China	JQ015297	40

### 2.3 Three‐dimensional structure prediction of the HN protein

The I‐TASSER (https://zhanggroup.org/I‐TASSER/) predicted the 3D structure of the HN protein isolated in our study. This server uses LOMETS (Local Meta‐Threading Server) to stimulate the 3D structure of candidate proteins. The I‐TASSER server used different 3D structures from those reported in the PDB (e.g. 1USR, 3T1E, 1E8U and 5B2C) for threading by the query amino acid sequence. In addition, this server used 10 other protein structures originating from multiple viruses with HN protein from the PDB and finally predicted 5 separate models. Here, we selected the 3D structure with the highest *C*‐score (confidence score) for estimating the quality of predicted models. For further analysis, we considered the 3D structure with a higher value of the *C*‐score, which revealed a high degree of confidence.

### 2.4 Analysis of molecular dynamic simulation

The GROMACS package (Versions 2019.4) is employed for molecular dynamic analysis in 50 nanoseconds (ns) to determine the structural stability and variations of the final HN 3D model. First, the amber03 force field was used to generate the topology for the HN structure. Then, the structure was positioned inside a TIP3P water box. Canonical NPT and NVT ensembles were utilized for 100 ps at a time step of 2 fs in the pressure and temperature ranges of 1 bar and 300 K, respectively, to equilibrium all MD simulation systems. Finally, MD simulations were performed at a time step of 2 fs in a time step of 50 ns. The root‐mean‐square deviation (RMSD) was calculated at this time. The PyMOL software plotted the final molecular graphics.

### 2.5 Prediction of linear and conformational B‐cell epitopes

The two main types of B‐cell epitopes are linear and conformational epitopes. The linear epitopes appear continuously, whereas conformational epitopes are composed of a group of long‐distance amino acids. To predict the HN linear B‐cell epitopes, we used the Bepipred Linear Epitope Prediction 2.0 server (http://tools.iedb.org/bcell/). In addition, the Ellipro server (http://tools.iedb.org/ellipro/) was applied to predict both linear and conformational epitopes for antibodies. The Ellipro server, for its analysis, needs the 3D structure of the HN protein in PDB format.

## 3 RESULTS

### 3.1 HA

The isolate of NDV reported here was collected from birds with clinical signs of northeastern Iran in 2016. The strain was assessed using an HA. This test indicated that the sample appeared to have a positive result. In our previous study (Tavassoli et al., 2019), we were shown that this sample was placed in the velogenic category.

### 3.2 Phylogenetic analysis of the HN

**FIGURE 1:**
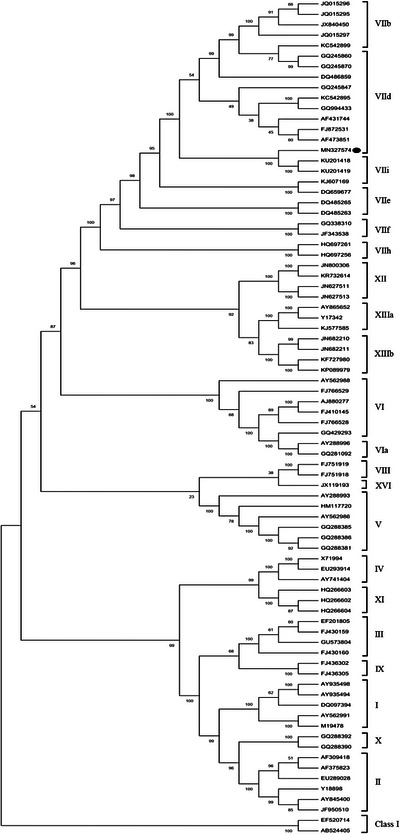
Phylogenetic tree of Newcastle disease virus (NDV) strains based on the nucleotide sequence of the haemagglutinin–neuraminidase (HN) gene (entire ORF; 1–1716 nucleotides). The provisional designations of the genotypes or subgenotypes are indicated on the right. The neighbour‐joining method generated the phylogram with 1000 bootstrap replicates using MEGA X software. The accession number MN327574.1, marked by the bullet, is studied in this work (isolated from the northeast of Iran).

The HN nucleotide sequence of the isolated NDV was registered in the GenBank database under the accession number MN327574.1. Using MEGAX software (version X) and 1000 bootstrap replicates, a phylogenetic tree (Figure 1) was constructed on the complete CDS of the HN gene sequences of 83 NDV isolates corresponding to different genotypes around the world, and commercial vaccine strains including accession numbers: AF077761 (LaSota), AF309418 (B1), M19478 and AY562991 (Ulster). The comparative analysis was subjected to the nucleotide sequence spanning from 1 to 1716 bp. The GenBank accession numbers and the country of origin of nucleotide sequences of the HN gene along with our sequence (shown by the bullet in Figure 1) were used for the phylogenetic analysis shown in Table 2. Moreover, in this table, we list the genotype and subgenotype of each isolate. The clustering patterns of our isolate revealed that its grouping is in class II, genotype VII and subgenotype VIId. Moreover, the phylogenetic analysis suggested a close relatedness between the study isolate and strains previously reported from Iran (KU201818, KU201419). These results showed the predominant circulation of genotype VII NDV in Iran. Moreover, homology analysis showed that the isolated strain has similarity score between 95% and 99% to compare with HN amino acid sequences of other NDV strains isolated from different regions of Iran (Figure S1).

### 3.3 Investigation of variations in HN coding sequence

To analyse variations among different NDV genotypes and NDVII subgenotypes, we predicted the coding sequence of HN genes in diverse isolates using the CLC software. Then, the predicted coding regions are aligned to determine the amino acid substitutions throughout the HN protein sequence, particularly at critical sites such as receptor binding, fusion promotion, sialic acid binding and antigenic regions.

After sequence comparisons of different NDV genotypes, it is shown that the length of the HN protein of NDV is variable between 570 and 616 amino acids (Khattar et al., 2009; Liu et al., 2019; Jakhesara et al., 2016). Here, our HN protein isolate contained 571 amino acids, which is one of the main properties of the virulent NDV (Maminiaina et al., 2010). Therefore, this amino acid sequence is used for comparisons to find mutations and substitutions in several dominant regions, including receptor binding, fusion promotion, sialic acid binding and antigenic sites between different vaccinal strains and genotypes of NDV (Table 3). The vaccinal strains include Ulster, LaSota and B1. In addition, these comparisons were made to find variations among different genotypes of VII NDVs and our isolate (Table 4).

**TABLE 3:** Comparison of amino acid changes at the receptor binding site, fusion promotion region, sialic acid binding and antigenic sites of the haemagglutinin–neuraminidase protein sequences between all genotypes of Newcastle disease viruses (NDVs).

Antigenic sites			Sialic acid binding site 234–239						Fusion promotion region				Receptor binding site			Genotype	Country	Gene Bank NO.
494, 513–521, 569	345–353	193–201							145	142	140	127	526	416	401			
D, RITRVSSSS, D	PDEQDYQIR	LSGCRDHSH	S	C	S	K	R	N	A	V	L	I	Y	R	E	I	Ireland	AY562991 (Ulster)
G, RITRVSSS, D	PDEQDYQIR	LSGCRDHSH	S	C	S	K	R	N	A	V	L	I	Y	R	E	II	China	AY845400 (LaSota)
G, RITRVSSSS, D	PDEQDYQIR	LSGCRDHSH	S	C	S	K	R	N	A	V	L	I	Y	R	E	II	USA	AF309418 (B1)
A, RITRVSSSS, G	PDEQDYQIR	LSGCRDHSH	S	C	S	K	R	N	T	V	L	V	Y	R	E	III	China	FJ430160
D, RITRVSSRS, D	PDEQDYQIR	LSGCRDHSH	S	C	S	K	R	N	A	V	L	V	Y	R	E	IV	The Netherlands	AY741404
N, RITRVSSSN, D	PDEQDYQVR	LSGCRDHSH	S	C	S	K	R	N	T	V	L	V	Y	R	E	V	USA	GQ288381
D, RVTRVSSSS, G	PDGQEYQIR	LSGCRDHSH	S	C	S	K	R	N	T	V	L	V	Y	R	E	VI	Hungary	AJ880277
D, RVTRVSSSS, D	PDQQDYQIR	LSGCRDHSH	S	C	S	K	R	N	I	V	L	V	Y	R	E	VIId	Iran	MN327574 (studied isolate)
D, RMTRVSSSS, D	PDEQDYQIR	LSGCRDHSH	S	C	S	K	R	N	V	V	L	V	Y	R	E	VIII	China	FJ751919
D, RITRVSSSS, D	PDEQDYQIR	LSGCRDHPH	S	C	S	K	R	N	A	V	L	V	Y	R	E	IX	China	FJ436302
D, RITRVSSSS, D	PDEQDYQIR	LSGCRDHSH	S	C	S	K	R	N	A	V	L	I	Y	R	E	X	Iran	GQ288392
D, RITRVSSSS, G	PDEQDYQIR	LSGCRDHSH	S	C	S	K	R	N	T	V	L	V	Y	R	E	XI	France	HQ266603
G, RVTRVSSSS, D	PDEQDYQIR	LSGCRDHSH	S	C	S	K	R	N	T	V	L	V	Y	R	E	XII	Peru	JN800306
D, RVTRVSSSS, V	PDEQDYQIR	LSGCRDHSH	S	C	S	K	R	N	T	V	L	V	Y	R	E	XIII	India	KJ577585
D, RITRVSSSS, D	PDEQDYQIR	LSGCRDHSH	S	C	S	K	R	N	T	V	L	V	Y	R	E	XVI	Dominique Republic	JX119193

**TABLE 4:** Comparison of amino acid changes at the receptor binding site, fusion promotion region, Sialic acid binding site and antigenic sites of the haemagglutinin–neuraminidase protein sequences between genotype VII of Newcastle disease viruses (NDVs).

Antigenic sites			Sialic acid binding site 234–239						Fusion promotion region				Receptor binding site			Genotype/Subgenotype	Country	Gene Bank NO.
494, 513–521, 569	345–353	193–201							145	142	140	127	526	416	401			
D, RVTRVSSSS, D	PDQQDYQIR	LSGCRDHSH	S	C	S	K	R	N	I	V	L	V	Y	R	E	VIId	Iran	MN327574
D, RVTRVSSSS, D	PDEQDYQIR	LSGCRDHSH	S	C	S	K	R	N	I	V	L	V	Y	R	E	VIId	China	FJ872531
D, RVTRVSSSS, N	PDEQDYQIR	LSGCRDHSH	S	C	S	K	R	N	I	V	L	V	Y	R	E	VIId	China	AF473851
D, RVTRVSSSS, D	PDGQDQIR	LSGCRDHSH	S	C	S	K	R	N	I	V	L	V	Y	R	E	VIId	China	GQ245847
D, RVTRVSSSS, D	PDEQDYQIR	LSGCRDHSH	S	C	S	K	R	N	I	V	L	V	Y	R	E	VIId	China	GQ245860
D, RVTRVSSSS, D	PDEQDYQIR	LSGCRDHSH	S	C	S	K	R	N	I	V	L	V	Y	R	E	VIId	China	GQ245870
D, RVTRVSSSS, D	PDEQDYQIR	LSGCRDHSH	S	C	S	K	R	N	I	V	L	V	Y	R	E	VIId	China	DQ486859
D, RVTRVSSSS, D	PDEQDYQIR	LSGCRDHSH	S	C	S	K	R	N	I	V	L	V	Y	R	E	VIId	China	AF431744
D, RVTRVSSSS, D	PDKQDYQIR	LSGCRDHSH	S	C	S	K	R	N	I	V	L	V	Y	R	E	VIIj	China	JQ015297
D, RVTRVSSSS, D	PDKQDYQIR	LSGCRDHSH	S	C	S	K	R	N	I	V	L	V	Y	R	E	VIIj	China	JQ015295
D, RVTRVSSSS, D	PDKQDYQIR	LSGCRDHSH	S	C	S	K	R	N	I	V	L	V	Y	R	E	VIIb	China	KC542899
D, RVTRVSSSS, D	PDKQDYQIR	LSGCRDHSH	S	C	S	K	R	N	I	V	L	V	Y	R	E	VIIb	China	JX840450
D, RVTRVSSSS, D	PDKQDYQIR	LSGCRDHSH	S	C	S	K	R	N	I	V	L	V	Y	R	E	VIIb	China	JQ015296
D, RVTRVSSSS, D	PDQQDYQIR	LSGCRDHSH	S	C	S	K	R	N	I	V	L	V	Y	R	E	VIIi	Iran	KU201418
D, RVTRVSSSS, D	PDQQDYQIR	LSGCRDHSH	S	C	S	K	R	N	I	V	L	V	Y	R	E	VIIi	Iran	KU201419
D, RVTRVSSSS, D	PDEQDYQIR	LSGCRDHSH	S	C	S	K	R	N	I	V	L	V	Y	R	E	VIIe	China	KJ607169
D, RVTRVSSSS, D	PDEQDYQIR	LSGCRDHSH	S	C	S	K	R	N	I	V	F	V	Y	R	E	VIIe	China	DQ659677
D, RVTRVSSSN, D	PDGQDYQIR	LSGCRDHSH	S	C	S	K	R	N	I	V	L	V	Y	R	E	VIIe	China	DQ485265
D, RVTRVSSSS, D	PDGQDYQTR	LSGCRDHSH	S	C	S	K	R	N	I	V	L	V	Y	R	E	VIIe	China	DQ485263
D, RVTRVSSSS, G	PDEQDYQIR	LSGCRDHSH	S	C	S	K	R	N	I	V	L	V	Y	R	E	VIIf	China	GQ338310
D, RVTRVSSSS, G	PDGQDYQIR	LSGCRDHSH	S	C	S	K	R	N	I	V	L	V	Y	R	E	VIIf	China	JF343538
D, RVTRVSSSS, D	PDKQDYQIR	LSGCRDHSH	S	C	S	K	R	N	I	V	L	V	Y	R	E	VIIg	China	KC542895
D, RVTRVSSSN, D	PDGQDYQIR	LSGCRDHSH	S	C	S	K	R	N	I	V	L	V	Y	R	E	VIIe	China	DQ485265
D, RVTRVSSSS, D	PDKQDYQIR	LSGCRDHSH	S	C	S	K	R	N	I	V	L	V	Y	R	E	VIIg	China	GQ994433
D, RVTRVSSSS, D	PDEQDYQIR	LSGCRDHSH	S	C	S	K	R	N	T	V	L	V	Y	R	E	VIIh	Indonesia	HQ697261
D, RVTRVSSSS, D	PDKQDYQIR	LSGCRDHSH	S	C	S	K	R	N	I	V	L	V	Y	R	E	VIIj	China	JQ015295

Based on the previously published reports (Yan et al., 2020; Sabouri et al., 2018; Liu et al., 2015; Ke et al., 2010; Zaitsev et al., 2004; Li et al., 2015), the residues in positions 401, 416 and 526 are determined as receptor binding sites, and residues from 234 to 239 are known as sialic acid binding sites. For these two regions, there were no observed substitutions among different genotypes (Table 3). Nevertheless, several specific substitutions were found in the amino acids constitute up the fusion promotion and antigenic sites of the HN proteins. The fusion promotion region of the HN protein includes the amino acid numbers 127, 140, 142 and 145. Comparative analysis of the fusion promotion region revealed substitutions at amino acids 127 (I→V) and 145 (A→T/I/V). The proposed antigenic sites (Yan et al., 2020; Jakhesara et al., 2016) include residues 193–201, 345–353, 494, 513–521 and 569. In addition, we indicated several substitutions at positions 200 (S→P), 347 (E→G/Q), 349 (D→E), 352 (I→V), 494 (D→G/A/N), 514 (D→G/A/N), 520 (S→R), 521 (S→N) and 569 (D→G/V) within the antigenic sites. However, there was an amino acid substitution at position 347 in the amino acid residues from 345 to 353 at the antigenic site, which is known as the primary linear epitope of HN (Cho et al., 2007; Cho et al., 2008). It means that residue 347 in our studied strain is glutamine (Q), but in the other isolates, it shows variable residues such as glutamate (E) or glycine (G). Moreover, there is another obvious amino acid substitution in the antigenic site from 513 to 521 and at position 569 (Table 3).

The results of comparisons among different VII NDVs and our isolates at the key regions of HN protein are represented in Table 4. These analyses represented the main variations at 140, 347, 521 and 569. As a result, with the exception of residue 140, which is located in the fusion promotion region, the other three residues (347, 521 and 569) are positioned in the HN protein's antigenic site (Table 4).

### 3.4 Prediction of 3D structure and molecular dynamic simulation analysis

**FIGURE 2:**
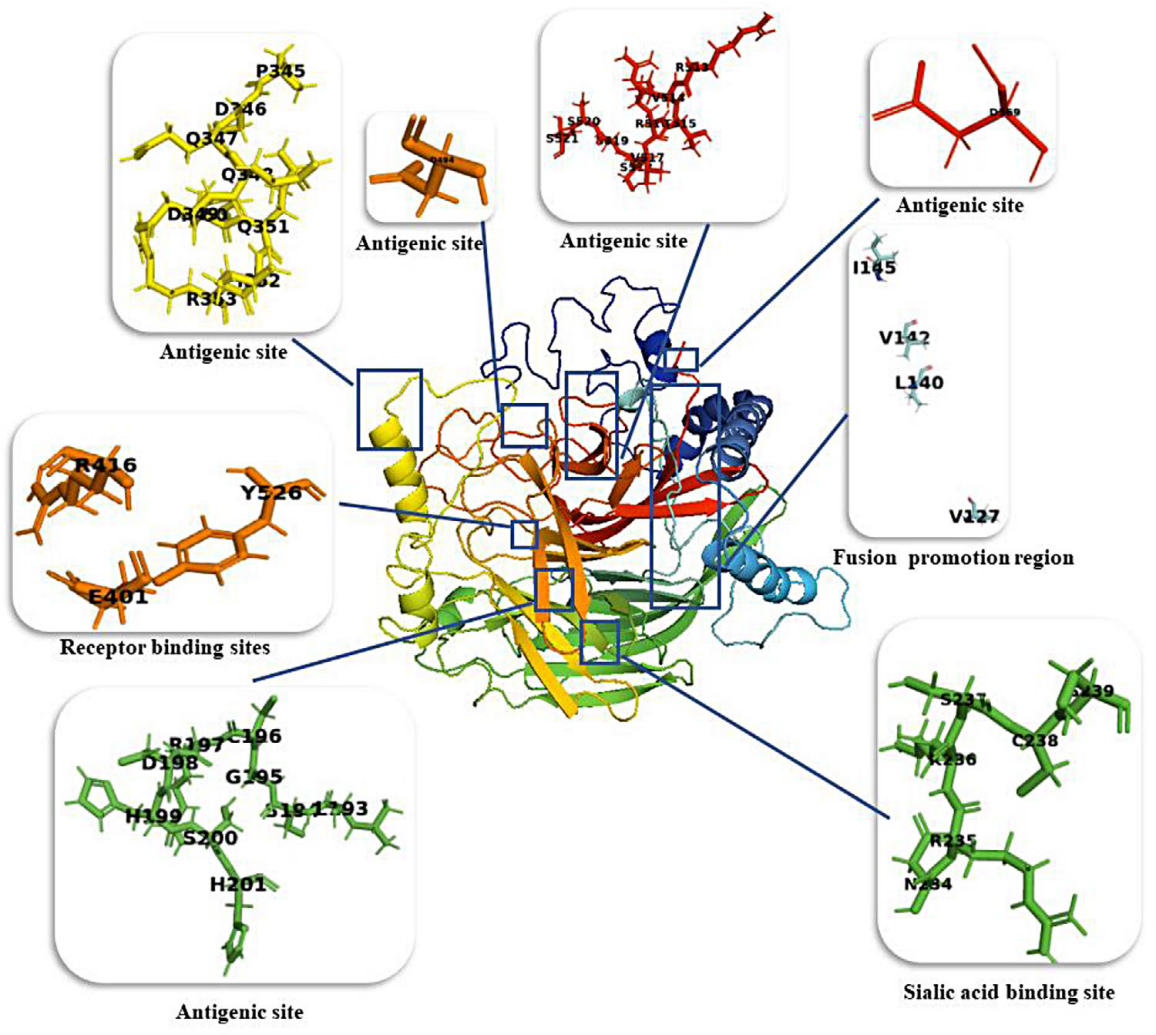
Three‐dimensional modelling of isolated Newcastle disease virus (NDV) haemagglutinin–neuraminidase (HN) protein (accession number: MN327574.1) with the residues at the receptor binding, fusion promotion, sialic acid binding and antigenic sites. The figure provides rainbow to have whole protein coloured accordingly N‐term is blue, C‐term is red, and middle colours vary.

In this work, the I‐TASSER server was used to generate the 3D structure from our isolated HN protein sequence (Figure 2). The *C*‐score for the selected model was −0.93. This score is typically in the range of [−5, 2], where a higher value signifies a model with increased confidence and vice versa. This model represents the positions of the desired residues at the receptor binding, fusion promotion, sialic acid binding and antigenic sites of our isolated HN protein. The positions of these residues for each site are represented in Figure 2.

**FIGURE 3:**
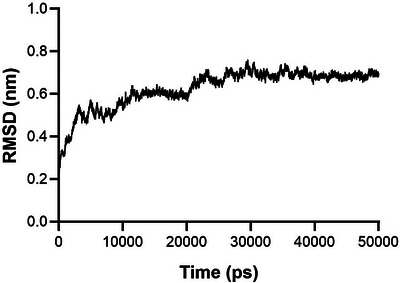
Result of molecular dynamic simulation analysis using the GROMACS software for root‐mean‐square deviation (RMSD) of the haemagglutinin–neuraminidase (HN) protein. The results for RMSD confirm that the simulation time (50 ns) is adequate to determine the structural stability and variations of the HN model.

The HN 3D model was subjected to the GROMACS software for molecular dynamic simulation analysis to determine the structural stability and variations of the PDB structure of HN protein. It is calculated by measuring the index of RMSD. Here, the molecular dynamic simulation was run for a duration of 50 ns. The results for the analysis of RMSD during this stimulation time are represented in Figure 3. Our findings for RMSD confirm that the simulation time (50 ns) is adequate to determine the structural stability and variations of the HN model (Figure 3).

### 3.5 HN protein antigenicity evaluation

**FIGURE 4:**
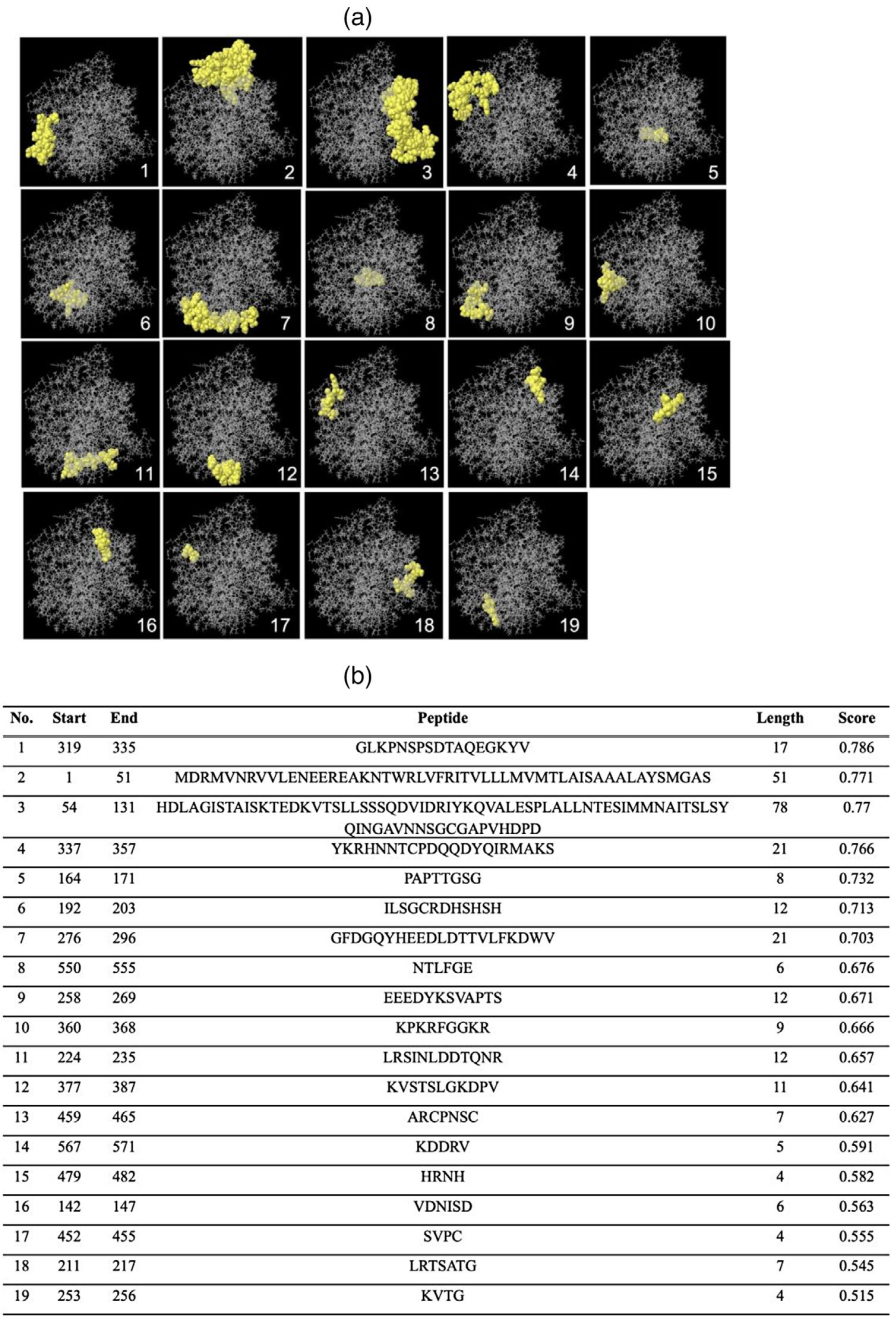
The predicted linear epitopes of haemagglutinin–neuraminidase (HN) protein by the Ellipro server. The epitope positions on the 3D structure and the specified information for each epitope are shown in (A) and (B), respectively.

**FIGURE 5:**
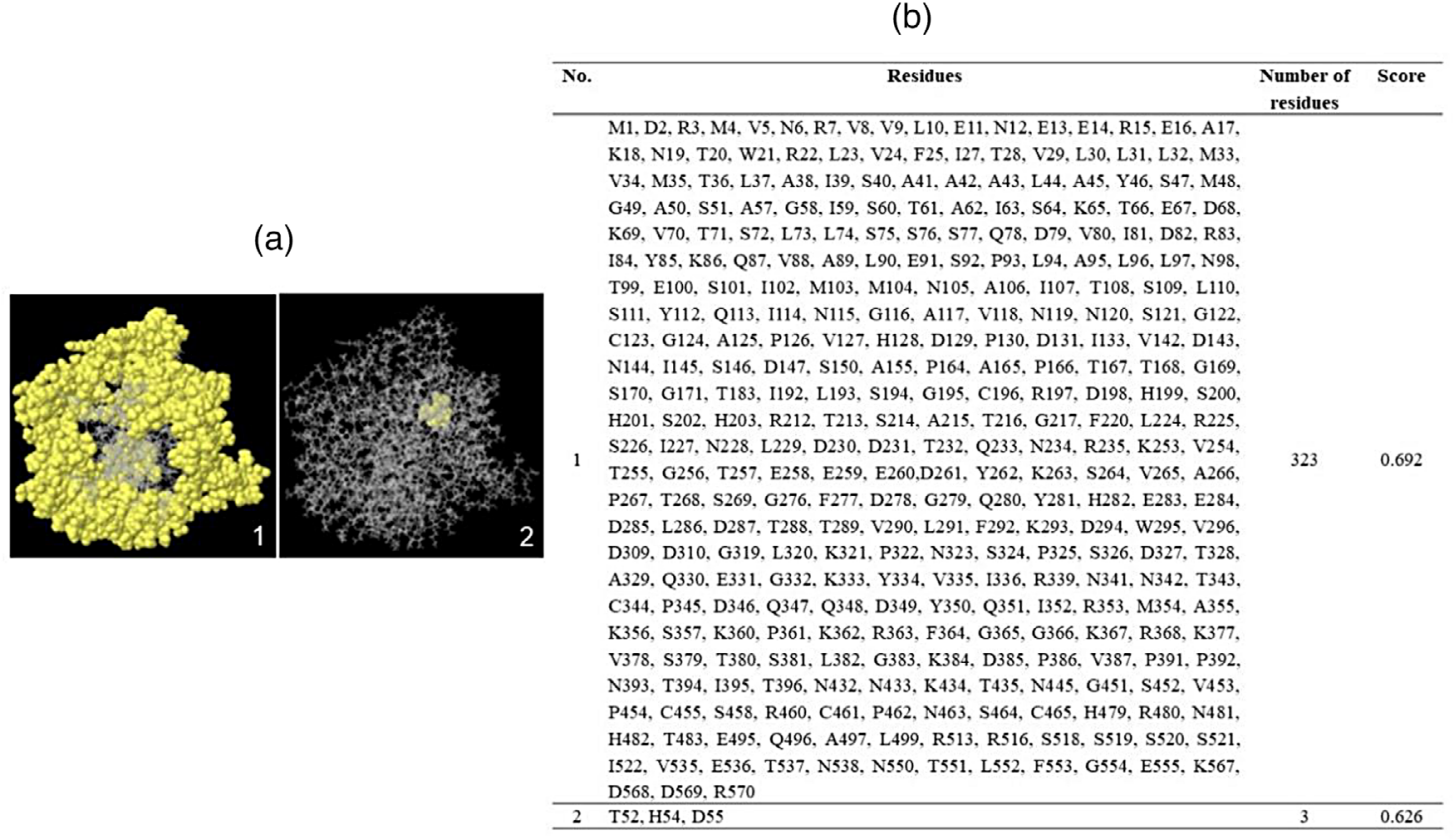
The predicted conformational epitopes of haemagglutinin–neuraminidase (HN) protein by the Ellipro server. The residue positions on the 3D structure and the specified information for each residue are shown in (A) and (B), respectively.

The BepiPred and Ellipro servers are utilized to investigate potential linear and conformational epitopes for B lymphocytes of the HN protein. We submitted the amino acid sequence and 3D structure to the BepiPred and ElliPro servers. First, the linear epitopes of the HN amino acid sequence (571 aa) were predicted by the BepiPred server. The sequences, the number of specified start and end residues and the length of each predicted epitope are listed in Table 5. The threshold is considered to be less than 0.5. Then, the positions and sequence information of all B‐cell linear epitopes predicted by the Ellipro server are depicted and listed in Figure 4A,B. Next, the antigenicity score ranges were calculated for HN and ranged from 0.786 to 0.515 (Figure 4B). Finally, we determined the conformational epitopes using the Ellipro server. In Figure 5A,B, all residues predicted to be conformational B‐cell epitopes are depicted. Here, a large number of HN residues have the potential to act as discontinuous epitopes with an antigenicity score ranging from 0.692 to 0.626.

**TABLE 5:** Predicted linear epitopes of haemagglutinin–neuraminidase (HN) protein by Bepipred server.

NO.	Start	End	Peptide	Length
1	5	18	VNRVVLENEEREAK	14
2	54	57	HDLA	4
3	64	83	SKTEDKVTSLLSSSQDVIDR	20
4	93	102	PLALLNTESI	10
5	112	140	YQINGAVNNSGCGAPVHDPDYIGGIGKEL	29
6	142	170	VDNISDVTSFYPSAYQEHLNFIPAPTTGS	29
7	196	201	CRDHSH	6
8	216	217	TG	2
9	228	235	NLDDTQNR	8
10	256	267	GTEEEDYKSVAP	12
11	281	292	YHEEDLDTTVLF	12
12	321	364	KPNSPSDTAQEGKYVIYKRHNNTCPDQQDYQIRMAKSSYKPKRF	44
13	380	385	TSLGKD	6
14	433	458	NKTATLYSPYKFNAFTRPGSVPCQAS	26
15	460	460	R	1
16	479	482	HRNH	4
17	495	499	EQARL	5
18	517	523	VSSSSIK	7
19	552	554	LFG	3

## 4 DISCUSSION

It has been confirmed in previous studies that there are conserved residues in the HN protein sequence that guarantee the correct function for receptor recognition, antigenic, NA and fusion promotion activities (Yan et al., 2020; Khattar et al., 2009; Yuan et al., 2011). For example, seven overlapping antigenic sites exist in the HN protein, whereas residues 345–353 form the only linear epitope in the HN structure susceptible to immune pressure. These residues play a significant role in generating and intensifying antigenic variation (Cho et al., 2007, 2008; Zhu et al., 2016; Li et al., 2015). The other conserved residues at key positions of the HN protein sequence, which drive different biological functions, have been highlighted by transfecting the plasmids that harboured mutations in constructs that express the HN protein (Khattar et al., 2009). Several studies discovered that the tropism and virulence of NDV viruses varied depending on the type of HN gene sequence. It is proved by exchanging the HN genes among different strains of NDV and changing the primary conserved residues that they contributed to various biological activities. However, there were conflicts in some of the results of these studies (Jin et al., 2017; Huang et al., 2004; Khattar et al., 2009). The primary targets of a host's immune response to ND are HN and F proteins, so these two proteins have been considered the main objectives for developing several ND vaccines (Yan et al., 2020; Tavassoli et al., 2019; Izquierdo‐Lara et al., 2019).

In this study, we present a comprehensive analysis of the ND virus's HN protein isolated in Iran. After sequencing and finding the full‐length sequence of HN, this is used for phylogenetic analysis. The results showed that our isolate belongs to the VIId subgenotype (Figure 1, Table 2). Then, we compared amino acid variations at the receptor binding, fusion promotion, sialic acid binding and antigenic sites of the HN protein between all genotypes of NDVs (Table 3) and other more prevalent VII subgenotypes and vaccine strains (Table 4). The results of our comparisons among different genotypes and vaccinal strains demonstrated that the residues in the receptor and sialic acid binding sites are the same. However, there were differences in residues 127 (IV) and 145 (AT/I/V) in the fusion promotion region. The previous research suggested that the NDV HN protein seems to have at least five antigenic sites related to epitopes, including residues 193–201, 345–355 and a C‐terminal region comprising residues 494, 513–521 and 569 (Cho et al., 2007, 2008; Sabouri et al., 2018). In addition, the HN glycoprotein's amino acid residues 341–355 were also identified (Ke et al., 2010). Here, at the antigenic sites, as the most critical residues of the HN protein, there were multiple substitutions, but the main residues are 347 and 569, which showed variations (Table 3). We discovered significant differences among different VII NDVs and our isolates at residues 140, 347, 521 and 569. The most significant variations are positioned in antigenic sites with residues 347, 521 and 569. The residue 140 placed in the HN protein's fusion‐promoting domain (Table 4). These results demonstrate that the most critical sites in the HN protein of the ND virus that could be under mutation pressure are antigenic residues (Cho et al., 2007, 2008; Sabouri et al., 2018). However, the antigenic regions in our isolate are very similar to the other VIId subgenotypes of NDV isolated from different geographical places.

Furthermore, the results of 3D structure and molecular dynamic simulation for determining the RMSD of the HN protein led to the introduction of a stable structure with antigenic surface display potential (Figures 2 and 3). The prediction of linear and conformational B‐cell epitopes is guided by the antigenic residues that have been investigated previously (Cho et al., 2007, 2008; Li et al., 2015). Here, for our isolate, by employing the amino acid sequence and final 3D structure of HN protein, we presented lists of linear and conformational antigenic regions with their positions and scores (Table 5; Figures 4 and 5). In this work, we listed the antigenic sites of the HN protein in Tables 3 and 4 based on previous related scientific reports. We found that nearly all of the antigenic sites of HN (Tables 3 and 4) were present in the lists of potentially linear and conformational antigenic epitopes retrieved from online servers (Table 5; Figures 4 and 5).

By considering these potentially antigenic regions, it will be possible to design and develop a new generation of candidate vaccines to eliminate the ND virus globally. In particular, HN proteins are on the surface of NDV and play critical roles during host receptor binding and viral infection. Therefore, the production of antibodies against HN proteins is crucial for host protection.

It still needs to be improved upon after decades of research and development to create the perfect vaccine. On the other hand, a more potent vaccination can be created faster and at a cheaper cost by using in silico technologies.

This study employs immunoinformatic methods to assess the properties of antigenic HN protein to elicit an effective immune response. The current study is the exclusive result of a computer‐based computational method; to elicit the safety and efficacy of the vaccine, experimental validation on the immunogenic protein is required, which can include comprehensive in vivo and in vitro assessments. Thus, exploring and identifying the critical sites of HN protein will provide valuable information for designing and developing a new generation of attenuated vaccines against the circulating NDV strains (Yan et al., 2020; Triosanti et al., 2018; Li et al., 2015; Habib et al., 2018).

## 5 CONCLUSION

Bioinformatics has developed various tools for identifying and predicting epitopes and potentially antigenic regions within viral and bacterial infectious proteins (Soleymani et al., 2020). Immunoinformatics studies to find immunogenic regions in veterinary viruses result in the development of new vaccines that may be used instead of the conventional platform based on embryonated eggs. Evaluation of the antigenicity for predicting linear and discontinuous epitopes demonstrated significant potential to generate vaccines economically and quickly. Because the HN protein is one of the main immunogenic factors in the ND virus, targeting the antigenic regions can probably be a suitable option for developing a new generation of vaccines using rational vaccine design, protein and genetic engineering principles.

In conclusion, bioinformatics is a valuable tool for identifying potential neutralizing epitopes and guiding the selection of vaccine candidates. However, experimental validation remains a critical step in the vaccine development process.

## AUTHOR CONTRIBUTIONS

*Conceptualization; methodology; performed the analysis and wrote the manuscript*: Amin Tavassoli and Safoura Soleymani. *Supervised the project and revised manuscript*: Mohammad Reza Housaindokht. All authors read and approved the final draft of the manuscript.

## CONFLICT OF INTEREST STATEMENT

The authors declare that they have no conflicts of interest.

## FUNDING INFORMATION

Ferdowsi University of Mashhad

### ETHICS STATEMENT

None.

### PEER REVIEW

The peer review history for this paper is available at https://www.webofscience.com/api/gateway/wos/peer-review/10.1002/vms3.1491.

## Supporting information

Figure S1 Sequence alignment of the HN proteins from several different NDV strains in Iran.

## Data Availability

The authors confirm that the datasets used and/or analysed during the current study are available from the corresponding author on reasonable request.
