# Supplementary figures and images for: Nucleotide sequence characterization, amino acid variations and 3D structural analysis of HN protein of the NDV VIId genotype
Source: Vet Med Sci. 2024 Jun 21;10(4):e1491. doi: 10.1002/vms3.1491 (PMC11190948; doi:10.1002/vms3.1491)

**Supplement 1:** Sequence alignment of the HN proteins from several different NDV strains in Iran.


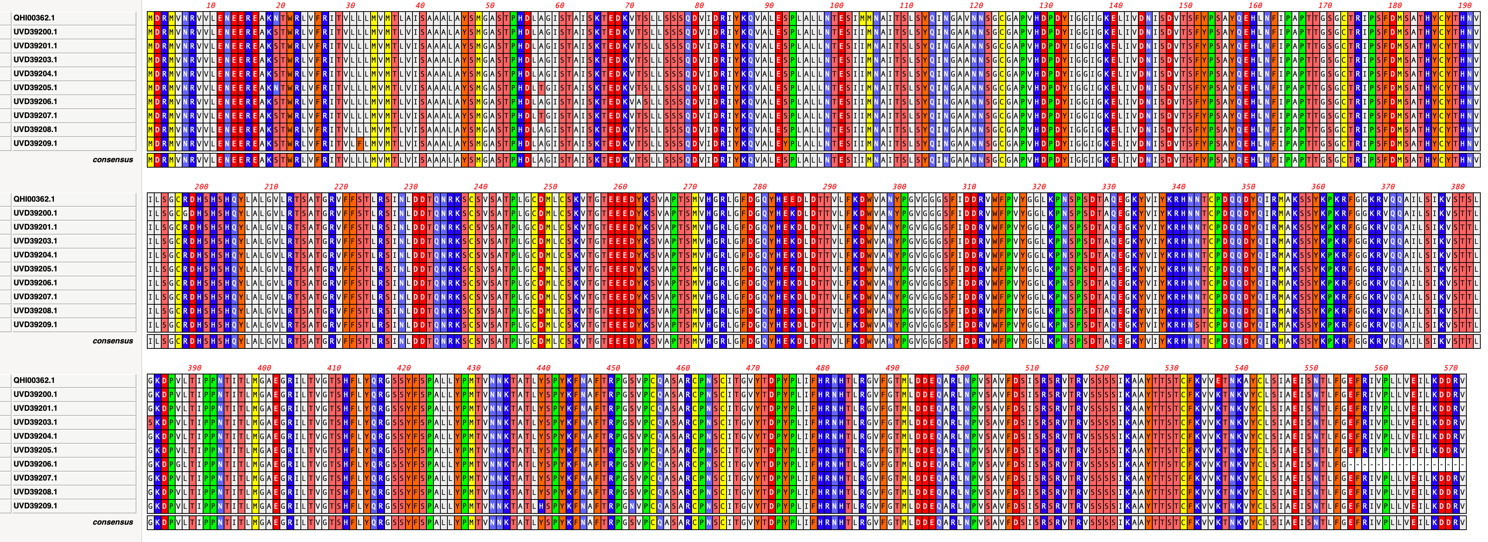

Supplement: Supplementary file 1 — Figure S1 Sequence alignment of the HN proteins from several different NDV strains in Iran. [file VMS3-10-e1491-s001.docx]
